# Hepatitis B screening in the Turkish-Dutch population in Rotterdam, the Netherlands; qualitative assessment of socio-cultural determinants

**DOI:** 10.1186/1471-2458-9-328

**Published:** 2009-09-09

**Authors:** Ytje JJ van der Veen, Onno de Zwart, Hélène ACM Voeten, Johan P Mackenbach, Jan Hendrik Richardus

**Affiliations:** 1 Department of Public Health, Erasmus MC, University Medical Center Rotterdam, Rotterdam, The Netherlands; 2 Division of Infectious Diseases Control, Municipal Public Health Service Rotterdam Area, The Netherlands

## Abstract

**Background:**

Hepatitis B is an important health problem in the Turkish community in the Netherlands. Increased voluntary screening is necessary in this community, to detect individuals eligible for treatment and to prevent further transmission of the disease.

**Methods:**

We investigated socio-cultural determinants associated with hepatitis B screening in male and female, first and second generation Turkish migrants, by means of Focus Group Discussions.

**Results:**

Socio-cultural themes related to hepatitis B screening were identified; these were social norm, social support, sensitivity regarding sexuality, reputation, responsiveness to authority, religious responsibility, cleanliness and religious doctrine regarding health and disease, and the perceived efficacy of Dutch health care services. Motivating factors were the (religious) responsibility for one's health, the perceived obligation when being invited for screening, and social support to get tested for hepatitis B. Perceived barriers were the association of hepatitis B screening with STDs or sexual activity, the perception of low control over one's health, and the perceived low efficacy of the Dutch health care services. Reputation could act as either a motivator or barrier.

**Conclusion:**

This study identified relevant socio-cultural themes related to hepatitis B screening, which may serve to customize interventions aimed at the promotion of voluntary hepatitis B screening in the Turkish-Dutch population in the Netherlands.

## Background

Hepatitis B is one of the major infectious diseases in the world [1]. It is a notifiable disease in the Netherlands, and each year around 1,800 hepatitis B virus (HBV) infections are reported nationally, of which 79% are chronic [2]. Chronic HBV infections cause 23% of all liver cancers in the Netherlands and are an important problem in ethnic minority groups, such as the Turkish community, which is the largest group of immigrants from newly or non-industrialized countries in the Netherlands [3,4]. The Turkish immigration in the Netherlands dates back to the sixties, when Dutch industries started to recruit abroad in order to attract extra labour forces. They targeted various countries of the Mediterranean. It was a joint effort of several Western European nations. As a result, between the end of World War II and the mid-seventies, guest workers came to the Netherlands and other North European countries, departing from several countries of the Mediterranean, including Spain, Italy, Morocco and Turkey. What was first meant to be a temporary migration turned into a permanent stay, as many immigrants settled in the Netherlands and later had their families come over for reunification [5,6]. While the Turkish community represents 7.7% (45,415 persons) of the total city population in Rotterdam, it accounts for 30% of reported chronic HBV infections [7-9]. Seventy percent of all infections (i.e. acute and chronic infections) in the Turkish-Dutch population involve people aged between 16 and 40. In this group, the mean incidence of reported HBV infections is 122 per 100,000 Turkish-Dutch individuals, much higher than the 35 infections per 100,000 persons reported in the total population of Rotterdam, and the 9 infections per 100,000 persons reported in the general Dutch population [2]. However, these figures underestimate the population-prevalence, because many chronic hepatitis B patients do not have symptoms of disease and are therefore not reported. Population-based studies indicate a prevalence of chronic HBV infections of 0.2% in the general Dutch population, and a much higher prevalence of 2.6 - 4.8% in the Turkish-Dutch population [4,8,10,11].

Transmission in migrant populations (such as Turkish migrants) in low-endemic countries (such as the Netherlands) likely reflects the transmission pattern of the country of origin; most individuals in the Turkish community have acquired HBV through vertical transmission (from mother to child during birth) [2]. However, in (young) adults the most important route of transmission is through horizontal transmission (i.e. sexual contact) [12]. A study in the Netherlands demonstrated that in 60% of the heterosexual cases of hepatitis B, the source of infection was a partner originating from an HBV-endemic region [13]. As 75-80% of the married Turks in the Netherlands are married to someone from Turkey (marriage migration) [14], the risk of horizontal transmission in the Turkish community is still high. It is estimated that immunization of persons with partners of non-Dutch nationality could prevent 36% of hepatitis B cases in heterosexuals [15].

Control of HBV infection presently focuses on screening pregnant women and vaccinating specific risk groups, such as newborns, children with a parent from an HBV endemic area (such as Turkey), and people with high-risk sexual behaviour [12]. Although these programmes are well-attended, there is no specific strategy for the detection and prevention of HBV in the adult Turkish-Dutch population. Screening for HBV should therefore be promoted in this group through public health interventions, in order to detect individuals eligible for treatment and to prevent horizontal transmission in sexually active and pre-active individuals. To develop these interventions, determinants of screening behaviour need to be identified. Studies of migrant groups in the USA have identified several behavioural factors that influence participation in HBV screening. These include the level of knowledge, attitude towards screening, perceived severity, perceived susceptibility, self-efficacy, cultural beliefs (e.g. traditional medicine), accessibility of health care, and demographic factors such as age, education, language proficiency, length of stay in the new country, having health insurance, and socio-economic status [16-25]. Studies into preventive behaviour (e.g. breast cancer screening) of Turks in either Turkey or the Netherlands report relevant determinants such as educational level, knowledge/former education about the disease, confidence, perceived susceptibility, seriousness, barriers and benefits [26-33]. Literature on the access of migrants to the Dutch health care system suggests that the most important barrier is communication between health care providers and clients [34]. Barriers in access and stigma have been reported by migrant black Africans with regard to HIV testing [35], but not with regard to HBV testing. Health insurance is obligatory in the Netherlands, and most health care costs will be re-imbursed by this insurance. However, this is often not the case for self-initiated hepatitis B testing and vaccination of adult migrants, as they are not formally defined as a risk group by the Ministry of Health. Therefore, the costs incurred might be a barrier for testing and vaccination in Turkish Dutch.

When health education places a strong emphasis on individual cognitive processes, and pays limited attention to the embeddedness of human health behaviour in cultural contexts and social structures, this may lead to low effectiveness of interventions. Therefore, basing interventions not just on behavioural constructs but also on socio-cultural factors, is expected to enhance the reception and appreciation by the public [36,37]. Anthropological and migrant studies revealed some plausible relevant socio-cultural determinants related to HBV screening [38-43]. These are social influences such as social norms and social support, and cultural aspects such as the sensitivity regarding sexuality, the importance of reputation, and responsiveness to authority. Also, as the majority of the Turkish-Dutch population is Muslim, religion may be an important determinant of screening through its doctrine regarding health and disease, religious responsibility, the concept of cleanliness, and of what is considered (un)lawful ('haram/halal'). To our knowledge, socio-cultural factors influencing the HBV-screening behaviour in the Turkish-Dutch population have not been investigated.

The aim of this study was to investigate behavioural and socio-cultural determinants associated with hepatitis B screening in the Turkish population in the Netherlands, in order to develop culturally appropriate interventions. The study applied a combination of qualitative and quantitative research methods, by means of focus group discussions and a survey. This paper reports the findings from the qualitative study, which aimed to obtain insight into socio-cultural determinants and underlying mechanisms that influence the enrolment in HBV-screening by the Turkish-Dutch population. Furthermore, we explored the relevance of these determinants in four subgroups distinguished by gender and migrant generation.

## Methods

### Participant selection

Our study included first and second generation migrants. First generation migrants (G1) were defined as persons born in Turkey, and having at least one parent who is born in Turkey as well. Second generation migrants (G2) were defined as persons born in the Netherlands, having at least one parent born in Turkey [7]. Third generation Turkish-Dutch, who are born in the Netherlands and who have at least one second-generation migrant parent, are a very small proportion of the total Turkish-Dutch population and are mainly children or adolescents [44]; therefore, they were excluded from our study. Discussions were organised separately for men and women, in order to secure a safe environment for sharing opinions. We expected that growing up in the Netherlands versus Turkey might influence the level of acculturation [45]. Therefore, we organised separate discussions for first generation migrants who emigrated before the age of 21 (G1A) and for those who emigrated at or after the age of 21 (G1B). Recruiting for G1A and G1B discussion groups was done with assistance of a local umbrella-organisation for Islamic organisations (SPIOR). This organisation contacted two different Turkish men and women associations. These associations are social groups that provide activities such as language courses, homework tutoring, and consultation in child raising. The leaders of these associations announced the planning of a group discussion about health issues, and asked the members of the associations to participate. Additionally, two G2 (i.e. second generation) discussion groups were held with students of a Regional Vocational Training Centre, as this was the most convenient location to speak with groups of younger people; recruiting was done by a study-coordinator who asked Turkish-Dutch students to participate. All participants voluntarily signed up to join the discussion group, and received a coupon as token of appreciation at the end of the discussion.

We originally planned to conduct six focus group discussions: G1A, G1B and G2 with men; and G1A, G1B and G2 with women. Five of those six planned discussion meetings succeeded, but we were unable to get enough participants for a male G1B group. Since we also realized that the focus group discussions had all been conducted in rather well-off suburbs, we then decided to organise two more focus group discussions with men and women in suburbs that were recently defined as 'disadvantaged' by the Ministry of Housing, Suburbs and Integration. Thus, in total seven group discussions were held.

### Data collection

The focus group discussions were led by male and female Turkish discussion leaders, and a Dutch female researcher. The latter person led the discussion with G2 women in the Dutch language. The other focus group discussions were conducted by a Turkish group leader in the Turkish language, except for the G2 male groups, which was led in Dutch by a Turkish discussion leader. The Turkish discussion leaders were less experienced in leading discussions than the Dutch researcher. They therefore attended a 2-hour training session, in which an information leaflet about Hepatitis B was discussed, and in which they were trained in moderating skills. During the discussions all leaders used a FGD guide. They also had a leaflet in which the information on Hepatitis B to be provided during the discussion was written out fully (see Appendix I). All male group discussions were led by a male discussion leader, and all women groups by a female leader. Demographic data of the participants were gathered by a short questionnaire at the start of the discussion. Each discussion was recorded except for one, because the tape recorder broke down at the start. During this meeting minutes were taken by a Turkish-Dutch observer, who was present in all meetings. As these observers would write verbatim transcripts of the recording afterwards, it was helpful for them to be present during the discussions. The focus group discussions were held over a period of two months (April-June 2007). The Medical Ethical Review Board of Erasmus MC, University Medical Centre Rotterdam, approved of this study.

### Procedures

Discussions started with broad questions about knowledge of hepatitis B, its prevention and the perceived risk of this disease. Next, we provided concise verbal information about HBV, transmission, testing and vaccination, whereby all routes of infection (from mother to child, through blood contact/wounds, and through sexual contact) were explained (see Appendix 1). As transmission of HBV in injecting drug users in the Turkish community is very rare [46], we decided not to mention this in the focus group discussions, but focussed on the main routes of transmission in this population. The group was then led into a brainstorm session, in which reasons for screening (or non-screening) and vaccination (or non-vaccination) were identified. In this way, lists of barriers and motivators related to screening and vaccination were composed on a large flip-over easel pad. After this brainstorm session, a short coffee break was held for the participants. During this break, the discussion leader summarized the socio-cultural topics following the themes that had been previously derived from literature: social norm, social support, sensitivity regarding sexuality, reputation, responsiveness to authority, religious responsibility, cleanliness, and religious doctrine regarding health and disease. The topic of the perceived efficacy of Dutch health care services was added to this theme list, as this was a new issue. After the coffee-break, the discussion leader elaborated with the participants on the items that were mentioned during the brain storm session in a systematic way, i.e. following these themes. On average, a focus group discussion lasted 90 minutes.

### Analysis

The group discussions were analysed using the approach as described by Krueger [47]. This approach includes the following steps: producing verbatim transcriptions, clarifying the transcripts by discussion, giving thematic labels to relevant sections, and summarizing the information. Verbatim transcriptions were noted down by the Turkish-Dutch observers, thereby directly translating the Turkish discussions into Dutch transcripts. The researcher and either the discussion leader or the observer then discussed the transcripts for clarification. Subsequently, the principal researcher analysed the transcripts by giving thematic labels, which were used in the summary during the discussion, to parts of the discussions. Next to the pre-defined themes, newly emerging themes could be defined. A second researcher labelled two of the transcripts independently. The two researchers then discussed the labelled transcripts, reaching a consensus on the emergence of themes. The other five transcripts were labelled according to this consensus by the principal researcher only. As during the discussion and analysis no clear differences were noticed between opinions from G1A and G1B groups, we combined these two groups into one overall first generation group for further analysis. After labelling, the key determinants in each theme were summarized by gender and generation. Finally, summaries of the discussions per theme, in which gender and generation were distinguished, could be composed. In this paper, we structure the themes into three realms that influence screening and vaccination for HBV, namely social, cultural and religious factors.

## Results

### Demographics

In total, seven focus group discussions were held. Age and generation of the participants are presented in Table 1. All parents of the 54 participants were born in Turkey. The majority of G2 participants were not married (82%) and did not have children (91%). G1 participants in most cases were married (78%) or widowed/divorced (6%) with children. Of the participants with children, 45% had children aged 16 and above (sometimes in combination with younger children). The majority of men (74%) reported to have a medium level education, two had university level education and four attended primary school only. Two women had not received any education and 32% attended only primary school, while the majority had a medium (45%) or higher (16%) level of education. The participants were asked to score their Dutch language proficiency (level 1-3/poor-excellent); the mean score for men was 2.14 while this was 2.06 for women. The groups from the disadvantaged suburb did not differ from the other groups regarding level of education and Dutch language proficiency. Most of the G1 participants were older compared to those in the G2 groups. To increase readability, we will sometimes use the terms 'older women' or 'older men' when reporting on G1 participants. For G2 participants we will sometimes use the terms 'young(er) men', 'young(er) women', 'boys' or 'girls'.

**Table 1:** Composition of the Focus Group Discussions regarding age and migrant generation

	**N**	**Age**			**Generation**		
			
		**Min**	**Max**	**Mean**	**1A^1^**	**1B^2^**	**2^3^**
Women							
group G1A	7	19	43	33.3	5	1	1
group G1B	8	26	69	44.8	0	7	1
group G2	8	17	22	20.0	2	0	6
low SES group^4^	8	25	47	32.9	5	2	1
Total	31				12	10	9

Men							
group G1A	9	36	76	46.2	4	4	1
group G1B	-	-	-	-	-	-	-
group G2	5	17	19	18.2	0	0	5
low SES group^4^	9	18	24	19.8	2	0	7
Total	23				6	4	13

^1 ^persons who emigrated from Turkey to the Netherlands before the age of 21

^2 ^persons who emigrated from Turkey to the Netherlands at or after the age of 21

^3 ^persons who were born in the Netherlands, having at least one parent born in Turkey

^4 ^persons living in disadvantaged suburbs

### Social factors

Determinants in the social realm that influence the screening and/or vaccination behaviour may be (1) the social perception of hepatitis B, (2) the social norm regarding screening, (3) the social norm regarding vaccination, and (4) the social support regarding HBV screening.

Regarding the **social perception of hepatitis B**, initially none of the groups expressed a negative feeling. G1 women mentioned:

"Hepatitis B is a subject that everyone has to deal with. It has to do with health, and how to control one's health. It's not something that affects one's honour".

Only when during the discussion it became clearer that HBV infection might be caused by sexual contact, the subject became more sensitive. The older women discussed the possibility of people talking negatively about them. In addition, the girls started thinking about sexual contact as a mode of transmission and became less confident about the acceptability of the disease. In most groups, it was expressed that there is little knowledge about the sexual mode of HBV transmission, and that this ignorance does prevent that people with hepatitis B are stigmatised. Illustratively, a participant in the G1 men expressed:

"People just do not know much about this disease. They simply come for a sick-call at home, and do not bother about it".

If the level of knowledge would increase, and it would become commonly known that HBV is a sexually transmitted disease, this might lead to social stigma. In contrast, the women from the disadvantaged area mentioned that everyone should be educated so there would be less social stigma surrounding HBV. G1 men and women compared the stigma surrounding AIDS and HBV, and expressed that HBV is perceived totally differently. They felt it was impossible to discuss AIDS with the wider social environment, while HBV was well discussable. The young men in the disadvantaged area, however, did initially associate HBV with AIDS, as "*you get it in the same way as AIDS*".

As for the **social norm regarding screening**, there were generally no objections to having a blood test. In fact, having a medical check-up (including broad-spectrum blood tests) when visiting Turkey seemed to be common practice, especially in the aging population. G2 men (and less strongly, G2 women) noted that it might be inefficient to have too many blood tests. The G2 men expressed:

"Well, you might not want to go for a test, because you just intend to live a healthy life. I am not doing a test, just like that. I will first have a look at myself: where did I go wrong, and primarily I will correct myself in that (risk) behaviour".

It was also noted that the G1 women resisted social norms whether or not people should have a HBV-test, illustrated by the following quote:

*"As long as I am confident about myself, the others are not important. If we constantly have to think about what others will say about us, we could not do anything with our lives! People will always talk. The more openly you discuss things, the less gossip there will be". *(G1 women)

Thus, while the existence and influence of social norms was acknowledged, it was also seen as something that should be fought. Men seemed to perceive little social influence, which became clear from expressions such as: "What other people think, does not affect me".

When the **social norm regarding vaccination **was discussed, all groups had difficulties finding reasons to object to vaccination.

*"If there is a good vaccination for this disease, that is the best action to take!" *(G1 women)

Vaccination was perceived as a positive action, and many even expressed the intention to get vaccinated and stimulate others to do the same.

**Social support regarding HBV screening **was extensively discussed by G1 women who spoke of themselves as supportive mothers, and to a lesser extent, supportive wives. The bond with their children was important; they gave the impression that they would support their children even though they might have contracted HBV by sexual contact (which is disapproved of).

*"I will ask them (the children) why they want to go for screening. I will just ask, not because I do not trust what they have done. Whatever has happened, if there is a risk for having contracted a disease, of course they should go for a test." *(G1 female participant)

In a less obvious way, this seemed also to be true for supporting their husband; if he planned to be screened this would be encouraged, but not without asking an explanation of the reason why he wanted to be tested. For themselves, the G1 women did not speak about support from their husbands or children. They seemed to be making independent choices about health screening, with a husband who was either indifferent or (distantly) supportive. The G2 women expressed that in general health issues, there was a lot of social support in Turkish families: in going for treatment, in being compliant with treatment, and by giving psychological support when ill. However, regarding the social support for HBV-screening we could distinguish three different opinions in the G2 women groups. First, some girls seemed to have an open relationship with their parents.

*"I can discuss anything with my parents, because they will always support me." *(G2 women)

Second, some girls considered a test for a sexually transmitted disease (STD) as a private matter, which they would not like to discuss with their parents. Should they really be ill, then they would expect the family to be supportive. Third, there were girls who perceived HBV infection mainly as an STD and strongly felt their parents' rejection of (extra- or pre-marital) sexual contacts. Although they might be supported in the end, the barrier for sharing this with their parents was very high and they were not sure about the reaction of the parents. The young men seemed to be less worried than the young women about rejection by their family.

*"Within Turkish families, there is a very strong bond. It is very difficult to break this bond. If you are in trouble like this (having HBV), they will not ostracise you. Even if you have done bad things... they will really regret it, it will break their heart, but they will not ostracize you because that is just not done." *(G2 male participant)

The quote makes clear that young men feel strongly supported by their family. However, in some cases they perceived social interference of the family as a stressor, as there might be so much concern that they feel overwhelmed by it. Social support in G1 men was shortly discussed, but neither receiving nor giving support regarding HBV-testing provoked discussion.

### Cultural factors

Issues related to the cultural realm that influence HBV-screening behaviour may be (1) the sensitivity regarding sexuality, (2) the issue of reputation and (3) the responsiveness to authority.

As for the **sensitivity regarding sexuality**, a shift was noted in the perception of HBV (testing) during the discussions: in most groups HBV infection was initially not seen as an STD and there was a positive attitude towards testing. Once aware of the routes of transmission (i.e. blood and sexual contact), the aspect of sexual transmission became the dominating issue, and social-cultural influences became apparent. However, when the discussions were summarized, participants seemed to return to their initial beliefs regarding HBV infection and testing. In all groups, sexuality was said to be a sensitive subject. G1 men mentioned as reason for not going for screening a sense of being ashamed for the suspicion of others (of having had extramarital sex). Even though they themselves would have a clear conscience, they still could be affected by this suspicion. The younger men seemed to be more confident about dealing with others' opinions about their sexual behaviour. The men from the disadvantaged area even mentioned that *"we (the men) don't have the problem of virginity"*'. When the groups talked about extramarital sex, it almost always considered men, not women (except young women who were sometimes thought to have sexual engagements too). G1 women did not seem to perceive infection through sexual contact as a personal risk, although they mentioned they could be indirectly affected by the sexual behaviour of their husbands. As discussed in the section 'social support', the sensitivity regarding sexual behaviour and HBV-testing was an important issue for G2 women.

Regarding the issue of **reputation**, the G1 men expressed that an established good reputation could protect them from social suspicion. If a man with a good reputation would have an HBV test, peers would look up to him in admiration because of his responsible action. That person might act as an example for others in that sense. The girls mentioned that if they would be blamed for bad behaviour by others, that would be terrible and a big shock for their parents. In G1 women groups, the issue of elderly people being ashamed for having HBV was mentioned. This was even apparent during the discussions; it was noticed that the older women were less open in sharing ideas about health and disease. Congruent with their remarks about social norms about HBV, women expressed that one's reputation should be less important than one's health, and that it should not be a hindrance to get tested.

In the group of the young women, the confrontation with a male physician was discussed, which seemed to be a barrier for some of the girls. A Dutch male doctor was preferred above a Turkish male doctor by some, while others saw doctors as professionals no matter ethnic background and gender. The young men in the disadvantaged suburb had strong opinions about the impact of having HBV (screening) on their reputation. When a young man would like to marry a girl, her parents might check his history. Either having the disease or having been screened, might be a blemish and a reason for not accepting him as future son-in-law. Not everyone in the group agreed with this opinion.

In order to explore further possible cultural influences, it was asked whether **responsiveness to authority **could be related to hepatitis B screening. Strikingly, in all groups the authority of the Municipal Public Health Services (MPHS) became the subject of discussion rather than the authority of community leaders or parents, as we had expected. Everyone agreed that when screening and/or vaccination would be obligatory, this would be a motivating factor for the Turkish community to comply. In some groups, it was expressed that an invitation would make it easier for individuals to participate in screening. Should they worry about their behaviour, then the invitation would release them from suspicion of the social environment.

*"Well it has a bit to do with taboo, but now we have discussed it, I can go for screening without getting into trouble." *(G2 male participant, disadvantaged area)

However, during the discussions on responsiveness to authority, almost all groups questioned the efficacy of the Dutch health care services. This new emerging theme was thoroughly discussed and the results are to be found under the heading 'Efficacy of Dutch health care services'.

### Religious factors

Issues related to HBV-screening that were mentioned by the participants were also in the realm of religion. These may be (1) responsibility for one's health, (2) the concept of cleanliness and (3) the Islamic doctrine regarding health and disease.

Regarding religious feelings of **responsibility for one's health**, all groups except the G1 women expressed the importance of their religion for the choices they will make regarding their health. Without probing, the responsibility for one's personal health and for the health of one's (future) partner, family and other persons in the social environment were mentioned as reasons for testing.

For the G1 men, a group in which a religious leader participated, the **concept of cleanliness **was seen as a condition for living 'halal' (lawful), and was presented as the solution for the prevention of HBV infection.

*"Our prophet says: cleanliness is half of the faith. If someone is not clean, he might not go to heaven. A person who lives according to the rules of our religion will be almost 100% sure of not getting this disease *(HBV)." (G1 men)

This concept of cleanliness, living 'halal', includes hygienic cleanliness (washing hands, using own toiletries), but also not having extramarital sex, and seemed to be most poignant for both G1 and G2 men. In none of the women's groups were the words 'halal' or 'haram' (unlawful) mentioned.

Aspects of the **Islamic doctrine regarding health and disease **were discussed in the groups:

*"It is written: you have to do all you can in order to cure a disease"*. (G1 men)

Furthermore, it was noted that Muslims are obliged to care for the body, in order to be able to return it to Allah in an unblemished state. The young men mentioned religion also in the context of fate. They felt that there was a limit to what one could do to prevent disease, and that getting ill, in a sense, is also fate. However, their peers remarked that this might never be a reason for not trying your best to stay healthy. One group of young men also mentioned that in religion, there is always forgiveness and a solution for bad behaviour. This was also deemed valid in the case of extramarital sex, and for this group, this conviction would help them to speak with their parents about their (perceived immoral) behaviour. The G1 women did not connect their religion to their behaviour. When asked for the connection between their religion and having or preventing hepatitis B, the main focus in both groups was that extramarital sex is forbidden. The women did not mention that religion can prescribe to act positively for one's health, while all other groups did so.

### Efficacy of Dutch health care services

In almost all groups, the difference in quality of health care in Turkey and the Netherlands was discussed spontaneously. The participants felt that in Turkey there was more quality of care; doctors are willing to prescribe medication and to order tests for patients readily, while in the Netherlands doctors seem to be resistant to do so. G1 women and G2 men most strongly expressed their dissatisfaction:

*"For 1 or 2 years, I am not seeing the GP anymore. If I have some complain, I think... well leave it, the only thing I will get is a painkiller. (...) I ask you: is that a way to be treated?!" *(G2 male participant)

*"It might be off-topic, but I really want you to write this down: in the Netherlands the health-sector is really badly organised. A human life is not truly valued." *(G1 female participant)

In the groups in the disadvantaged area, besides doubts about the quality of care, there was also distrust towards the Dutch health care system. This seems to be related to a general unhappiness about the Dutch government. It was expressed that one would rather spend his money on health in Turkey than in the Netherlands, as "*they *(the Dutch government) *just try to get money from me" *(G2 male participant). Also there were strong opinions about the role of the government in the case of hepatitis B prevention. A few times it was said:

*"If this is such an important disease why do we only hear about it now?" *(G1 women)

None of the groups saw practical barriers in the accessibility of the testing facilities at the Municipal Public Health Services, although high costs were mentioned as a possible obstacle.

## Discussion

Focus group discussions with members of the Turkish-Dutch migrant community in the Netherlands indicated that socio-cultural factors influence their HBV-screening behaviour. The importance of the various determinants of screening varied by gender and generation. Relevant motivating factors for all participants were feelings of (religious) responsibility for one's health, and the perceived obligation to go for screening when receiving an invitation from the Municipal Public Health Services. Second generation participants perceived social support as a motivating factor to get tested for HBV; however young women strongly wanted to avoid that their social environment would associate their HBV screening with STDs or sexual activity. A barrier for the second generation males was that they experienced their health as 'fate' rather than being in their own control. This lack of control over their health was also a relevant negative factor for the married women in both G1 and G2 groups, because infection was perceived to be dependent on their husbands' sexual behaviour. A factor that would motivate these women to be screened for HBV was their independence in making health decisions. Especially in first generation women and second generation men, the efficacy of the Dutch health care services was questioned and was deemed likely to discourage screening behaviour. An important factor for both first and second generation men was the importance of their reputation. Reputations could work in a negative way especially when reputation still needs to be established by the individual, and in a positive way when reputation already has been established.

### Strengths and limitations of the study

The strength of this study is its focus on central socio-cultural themes, which provides a deeper understanding of the community as a whole. But this study also had several limitations. A general limitation of focus group discussions is that they may present a picture of what is socially acceptable in a community, rather than what is actually believed or is actually happening in a community. Second, as recruiting was done by volunteers in an informal setting, in almost all groups a few members of other subgroups took part. However, we tried to ensure that the majority of the participants met the criteria for inclusion in the particular discussion group. Third, another possible limitation of this study is that recruitment of participants was done by an Islamic organisation and by a Regional Vocational Training Centre. This way of recruitment may have caused selection bias, for instance the first recruits being more actively involved in religion than the latter. In reality however, more than 90% of Turkish Dutch identify with Islam [48], and a large proportion of this group is involved in both social-religious and secular activities (such as school and work). Therefore, recruitment by these different organisations may not have caused much differentiation between the groups. Fourth, in our study most G1 participants were older and married with children, while G2 participants were younger and often had a child-role in their family. As in the Turkish-Dutch population there are also G2 men and women who are married and have children, this should be taken into account in interpreting the results for G2 participants. Fifth, in the general Turkish-Dutch population, 30% only attended primary school, 54% has a medium level education, while 16% has a high education level [7]. Thus, the proportion of men with a medium level of education was larger in our study (74%), possibly due to greater interest of higher educated individuals in participating in discussion groups. Therefore, care should be taken in extrapolating the findings of this study to the whole of the Turkish community. Sixth, the focus group discussions with the G2 women were led by the Dutch principal researcher, whereas all other groups were led by Turkish discussion leaders. This might have influenced the level of openness and social desirability. Most of the discussions were led in the Turkish language. Indeed, the majority of the first generation participants would not have been able to participate in a Dutch-language discussion. A disadvantage of this was that there was not enough elaboration on certain concepts (e.g. 'halal'), because there was a quick common understanding between the Turkish participants and Turkish discussion leaders. For a thorough understanding of these concepts in relation to the willingness to go for screening, deeper exploration is necessary. Discussion leaders should be more specifically trained to overcome this problem. Seventh, as there was little knowledge about HBV, we provided *each group with the same information *about HBV and its prevention. During the discussion however, participants may not have fully absorbed the information and thought through all the implications of testing. In addition, the participants were discussing a hypothetical situation: if they would go for screening, what would be motivating or barriers? They were asked to think about a situation they did not know, which may have led to incomplete results. Last, because of a lack of capacity, Dutch transcripts of the Turkish discussions were not back-translated. This may have caused translation-related bias, such as an incorrect understanding of the observers of what was actually said. The observers however, were also of Turkish Dutch origin. We expect that the level of translation-bias was limited, because of their ability to understand both languages and their understanding of the discussed topics due to their actual presence during the discussion.

### Social factors

Social norms have the largest impact on G2 women, who seriously doubt their ability to speak to their parents about HBV (in the sense of it being an STD). This is understandable, because of Islamic doctrine regarding honour related to the sexual behaviour of women in the family [43]. Studies into barriers to HIV testing have also found that fear of adverse consequences, such as rejection or blame from the family, may cause women to reject a test [49]. Social support for the G1 women seemed not to be present. This is confirmed by findings from group discussions with Islamic Arabic migrant women in Sweden, who said: "it is we ourselves that must retain our health" and "I am the one helping all others" [50]. These opinions are also found in African migrant women in the US, with regard to their apprehensiveness to include their male partners in treatment decisions, or to bring them to the clinic for HIV testing [51]. In contrast with G1 women, G2 men and women do experience social support in health issues, especially from their parents. This family involvement on the one hand is appreciated as social support, but too much involvement and care may be experienced as social control and over-concern. This ambiguity is also reported by Islamic migrant women in the USA, who expressed that next to support, their families' involvement in doctor visits caused apprehensiveness about disclosing information that they would rather keep private [52]. Generally, all participants in our study said that their health prevailed over social, cultural and financial barriers. Two studies into Turkish women's ideas about health and sexuality have indicated that Turkish women do not only want to control the risk of shame for themselves, but also for the wider circle of social relationships. For many women, reducing the risk of STDs to protect their physical health introduces risks to their social relationships and to the well-being of their family and community. Thus, women place priority on the protection of their social health over their physical health [40,41]. These findings suggest that in our group discussions, especially women may have given socially desirable answers or were not aware of those subconscious influences on their decisions.

First generation women, and some second generation women, did not see themselves as being at direct risk for contracting HBV through sexual contact. The unmarried girls considered themselves as being not at risk, because of their virginity, and the married women believed their only risk factor was the sexual behaviour of their husband. In this way, prevention of HBV infection seemed to be either unnecessary or out of their control. The young women discussed their preference in visiting a doctor of either Dutch or Turkish nationality. Opinions differed, as some preferred a Dutch doctor, while others preferred a Turkish doctor, and some did not have a preference for either one. The young women in the aforementioned study in the USA strongly expressed a need for a Muslim doctor, having the perspective of an Islamic source that understands religious necessities [52].

### Cultural factors

Islamic countries have been described as being bureaucratic with a large power distance and a strong avoidance of uncertainty, which means an increased need for rules or regulations, in contrast to less discerning non-bureaucratic countries such as the Netherlands [53]. Turkish families are described as being authoritative households, with patriarchal family relationships. Especially between fathers and children, rules of conduct warrant a certain respect [39]. For this reason, the issue of responsiveness to authority was raised in the group discussions. Contrary to our expectations, the relationship between government and citizens was mentioned instead of the hierarchical relationships in families. Participants indicated that a personal invitation by the MPHS would motivate people to go for screening. This seems to contradict the perceived low efficacy of the Dutch health care services by this population, and other migrant groups [34]. It is not clear how this will impact participation in screening.

### Religious factors

Religion has been shown to be associated with health behaviours [54]. It is shown that religious salience, attending religious services and participating in religious activities, is significantly related to the use of health screening [55]. Religious norms may also hinder participation in HIV testing [56]. However, most of the research into the influence of religion has been done in the USA and Canada, considering the impact of the western Judeo-Christian religious discourse. Studies into the influence of the Islamic religion on health or preventive services are rare [57]. The Islamic faith urges an active search for knowledge and health-promoting activities, and recommends those who are ill to strive to do everything to regain their health [38]. These issues were most valid for the male and G2 female participants of the group discussions in this study. They linked their religion to positive health action (such as screening), while G1 women tended to link religion merely to forbidden things (such as extramarital sex). Furthermore, the impact of religion on feelings of responsibility for the health of oneself and that of others was clearly expressed in this study. All men related prevention of disease to the need for cleanliness (living 'halal'). Literature on this topic points out that cleanliness has a hygienic and a symbolic aspect (restoring an inner balance) [43]. The young men also noted limitations in human actions with regard to health, as eventually health is a matter of fate. This is in line with the expressions of Arabs, who see doctors as instruments in the hands of Allah, who ultimately is the Curer [57]. This passive attitude towards health is also described elsewhere [43].

## Conclusion

This study explored socio-cultural determinants related to hepatitis B screening, and their relevance for male and female first and second generation Turkish migrants in the Netherlands. Motivating factors were the (religious) responsibility for one's health, the perceived obligation when being invited for screening, and social support in being tested for HBV. Perceived barriers were the association of HBV screening with STDs or sexual activity, the perception of low control over one's health, and the perceived low efficacy of the Dutch health care services. Reputation could act as either a motivator or barrier.

The findings suggest that participation in HBV-screening will increase if people receive a personal invitation from the MPHS. When developing an intervention aimed at the promotion of HBV-screening, it seems worthwhile to appeal to feelings of responsibility for one's own health and that of others, which were expressed by all groups. To overcome stigmatization of hepatitis B as being a sexually transmittable disease, emphasis should be placed on the most common route of transmission in the Turkish population, i.e. by blood contact during birth. Especially for young, unmarried women this will take away a major barrier in coming forward for screening. The intervention should also address the perceived lack of control over one's own health, by empowering people in showing how they can positively contribute to their own health, that of their family and wider community. Particularly for men, HBV screening should be advertised as a positive health act, which could even improve their reputation. Last, the perceived low efficacy of the Dutch health care services should be tackled by clearly explaining the screening procedures.

While this qualitative study provides useful insight in the socio-cultural determinants related to HBV-screening and their underlying mechanisms, quantitative confirmation of these findings is necessary. We therefore plan to conduct a survey which, together with the qualitative data from this study, will provide the basis for the development of a culturally-appropriate intervention aimed at the promotion of HBV-screening in the Turkish-Dutch population in Rotterdam, the Netherlands.

## Competing interests

This manuscript has not been published elsewhere and is not under submission elsewhere. There is no conflict of interest.

## Authors' contributions

OZ, JHR and JM made substantial contributions to the conception and design of this study and revised the manuscript critically. YV organised the Focus Group Discussions, analysed the data, and drafted the manuscript. YV and HV were involved in data-interpretation and in revising the manuscript. All authors read and approved the final manuscript.

## Appendix I Information provided during the Focus Group Discussions

### First block of information about hepatitis B

(provided after having explored what the participants already know about hepatitis B)

Hepatitis B is caused by the hepatitis B virus.

Many people in the world are infected with hepatitis B. It is a disease that is easily transferred from one person to the other. Hepatitis B occurs more often in Turkey and the Turkish community in the Netherlands, compared to the autochthonous population.

If a mother carries the virus, her child may be infected with the virus during or after birth. Infection may also occur from person to person by blood (razorblades, needles, wounds). The virus can also be spread by sexual contact between men and women (or men).

When the virus enters the body, the body can deal with it in several ways, depending on the age of the person and the immunity (i.e. the strength to fight diseases) of the body.

1) In children, the immune system is not yet fully developed. This implies that the body of a child cannot kill the virus and get rid of it. Therefore, in children the virus will often remain in their body for the rest of their lives. The virus in those children may be spread to other people by blood- or sexual contact, during the rest of their lives.

2) The majority of adults that get infected with the virus will not notice to have been infected. They will not experience any signs of the disease. They are able to kill the virus and also produce antibodies. The next time the virus will come into the body; those antibodies will immediately kill the virus. However, 5% of the adults that are infected will not be able to kill the virus and become a life-long carrier of the virus.

3) One in three people who are infected will indeed become sick. This is called 'acute hepatitis B infection'. Signs of disease are: fatigue, 'flu-like' symptoms, loss of appetite, nausea, vomiting, sometimes fever and sore joints. Sometimes these signs are followed by getting a yellow skin, dark coloured urine, and light coloured faeces. Often, these signs remain longer present in adults than in children. The total time of sickness may be from a few weeks up to 6 months. 1 in 1000 people with this disease may die. 5-10% of these people will remain carrier of the disease.

4) In 5 to 25 year, 15-25% of the people who carry the virus life-long will develop liver cancer or other serious liver disease.

### Second block of information about prevention

(provided after exploring the existing knowledge regarding prevention of hepatitis B)

In order to prevent hepatitis B there are two possibilities:

1. Things one should NOT DO

2. Things one should DO

Regarding things one should not do, it is important to avoid blood contact and unprotected sexual contact, because the virus may spread in these two ways.

Regarding things one should do, these are the following:

1) Screening. At the Municipal Public Health Services (MPHS), blood may be taken and tested for hepatitis B. In this way, the lab may find out whether someone has had the virus or maybe still has the virus in his (or her) body. In the first case, one is immune for the disease, in the latter case one is carrier of the disease. Another option is that someone has never been in contact with the virus. This person is then still susceptible to getting the disease, meaning that if the virus comes into the body, the person does not have specific immunity for hepatitis B and might get sick. The general cost incurred in the blood test is 26 euro.

2) Vaccination. Those people who have never been in contact with the disease may still get it. In order to prevent this, one may be vaccinated against the disease. This means that 3 protective injections are given in the upper-arm, in a time-period of 6 or 7 months. The cost incurred in vaccinations at the MPHS is 146 euro. After the three vaccinations, a last blood test is done in order to check whether the vaccinations have indeed caused protection in the body.

## Pre-publication history

The pre-publication history for this paper can be accessed here:


